# Supplementary material for: Seed-induced acceleration of amyloid-β mediated neurotoxicity in vivo
Source: Nat Commun. 2017 Sep 11;8:512. doi: 10.1038/s41467-017-00579-4 (PMC5594032; doi:10.1038/s41467-017-00579-4)
Supplement: Supplementary file 1 — Supplementary Information [file 41467_2017_579_MOESM1_ESM.pdf]

Description of Supplementary Files

File Name: Supplementary Information

Description: Supplementary figures

File Name: Peer Review File

Direct *in vivo* evidence for seed-induced acceleration of amyloid-β neurotoxicity

Ramona F. Sowade[1] & Thomas R. Jahn[1,*]

[1]Schaller Research Group at the University of Heidelberg and DKFZ, Proteostasis in Neurodegenerative Disease (B180), German Cancer Research Center, 69120 Heidelberg, Germany. *Current address: AbbVie Deutschland GmbH & Co. KG, 67061 Ludwigshafen, Germany. Correspondence should be addressed to T.R.J. (email: thomas.r.jahn@abbvie.com).

Supplementary Information

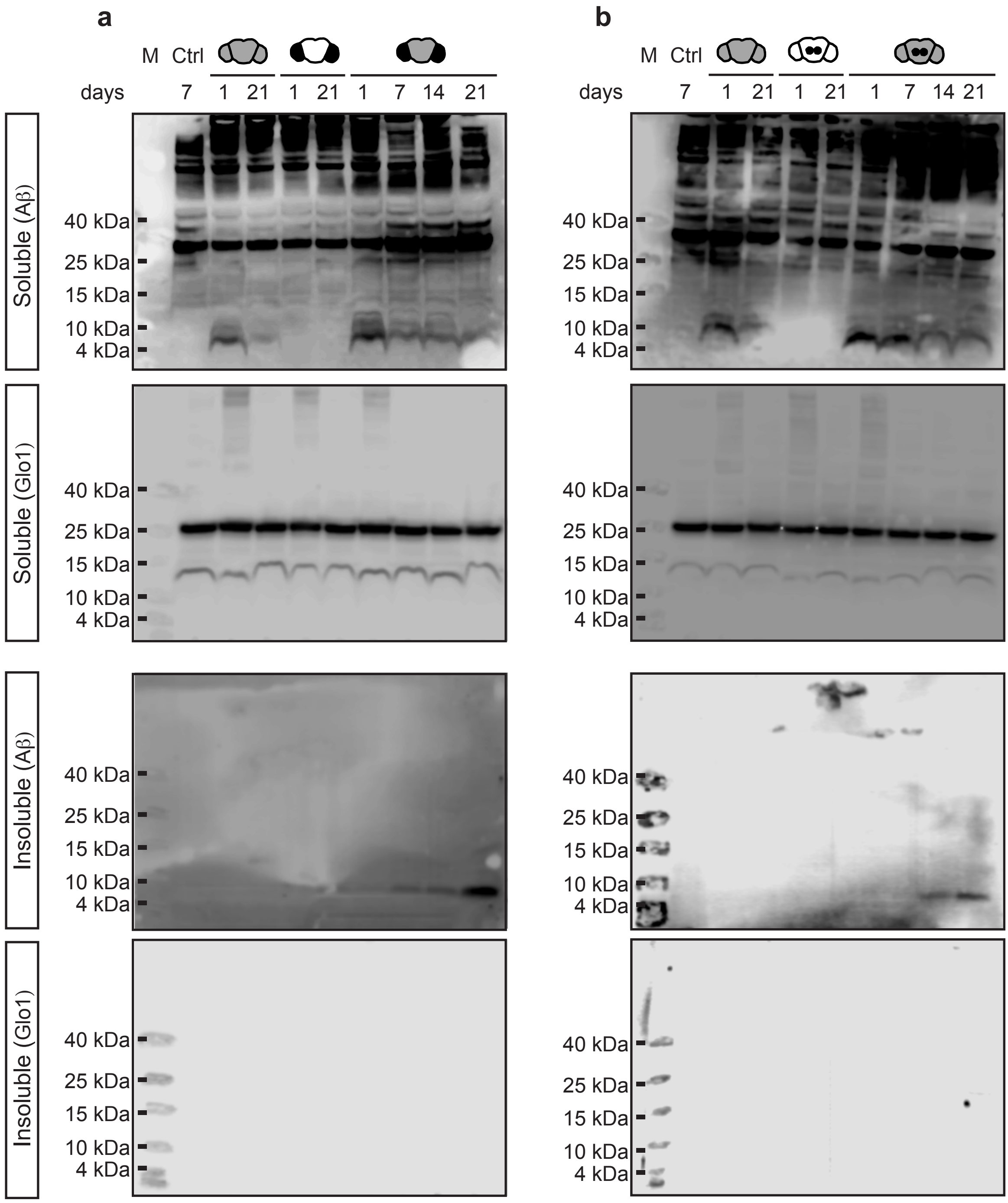

Supplementary Figure 1 | Time-dependent seeding of amyloid deposition in *Drosophila* brains. Exemplary western blot analysis of flies expressing either a single Aβ variant or the combination of $A\beta_{40}$ and $A\beta_{42}$arctic. The indicated time points (days post eclosion) were analysed. $A\beta_{42}$arctic seeds were either expressed in the optic lobes (**a**) or the central brain (**b**). Driver lines as indicated in Fig. 1. Total protein was extracted from the fly head and then seperated into soluble and insoluble fraction. The blots were probed with the monoclonal Aβ antibody 6E10 and with an antibody to detect Glyoxalase 1 (Glo1, loading control). The Spectra Multicolor Low Range Protein Ladder was used as size marker (M).

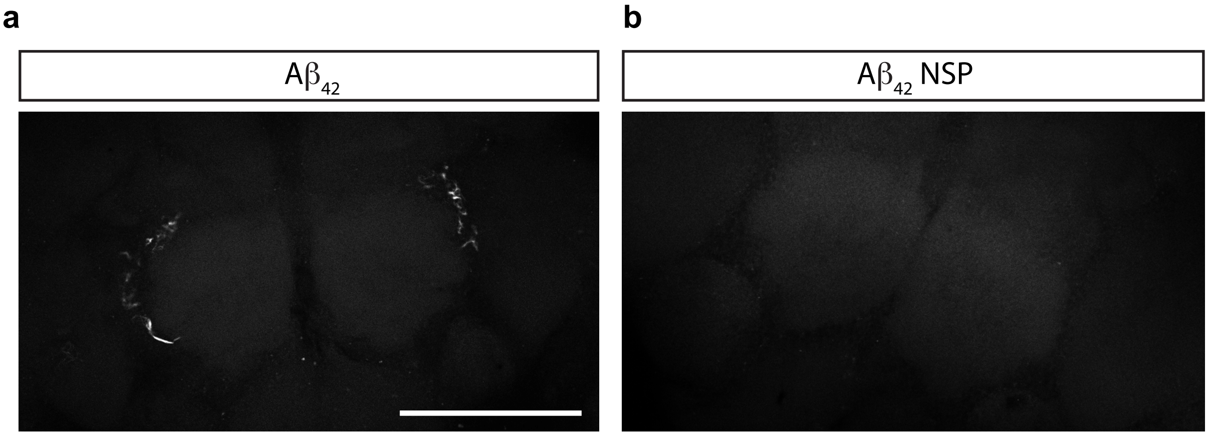

Supplementary Figure 2 | Aβ missing the secretion peptide does not accumulate in the brain. Immunohistochemical analysis of fly brains expressing $A\beta_{42}$ either with (**a**) or without (**b**, NSP) the secretion peptide. Transgene expression was driven using the *GMR14B06*-Gal4 driver. The confocal images show a zoom of the antennal lobes. Fly brains were stained with 6E10 detecting total Aβ. Only Aβ carrying the secretion peptide can be detected in the target region (a), whereas no 6E10 staining can be observed for $A\beta_{42}$NSP (b). Scale bar, 100 μm.

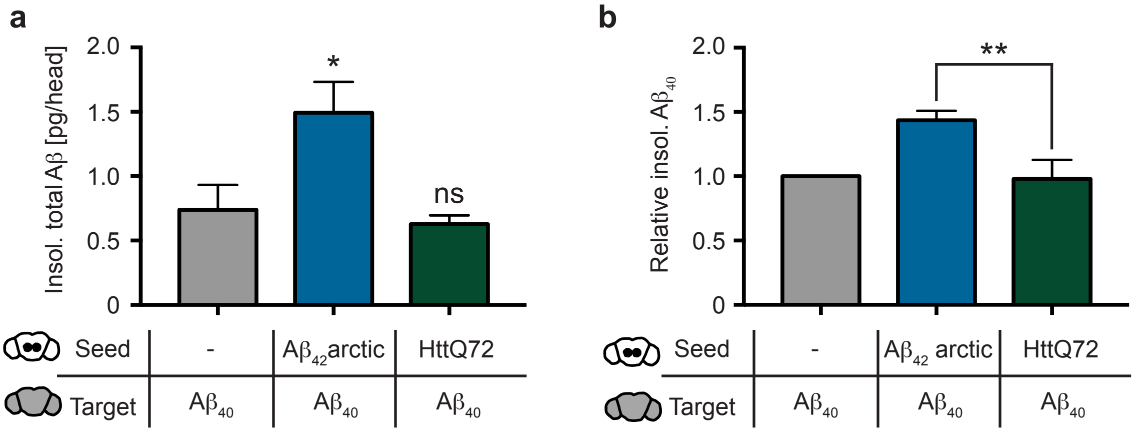

Supplementary Figure 3 | Aggregation-prone Aβ_{42}arctic but not Huntingtin seeds induce deposition of Aβ_{40}. Levels of insoluble Aβ in 21-day-old flies with the indicated genotypes were assessed using ECL analysis. As seeds we either expressed Aβ_{42}arctic or an aggregation prone Huntingtin variant with an expanded polyQ tract (HttQ72) in the central brain using *GMR14B06*-Gal4. (**a**) Level of insoluble total Aβ, i.e. the sum of Aβ_{40} and Aβ_{42} (error bars, s.e.m., n=5 independent biological replicates, one-way ANOVA (Dunnett's multiple comparisons test) in comparison to Aβ_{40} only (target) only, *P=0.0239). (**b**) Relative level of insoluble Aβ_{40} normalized to target only (error bars, s.e.m., n=4 independent biological replicates, one-way ANOVA (Dunnett's multiple comparisons test), **P=0.0094).

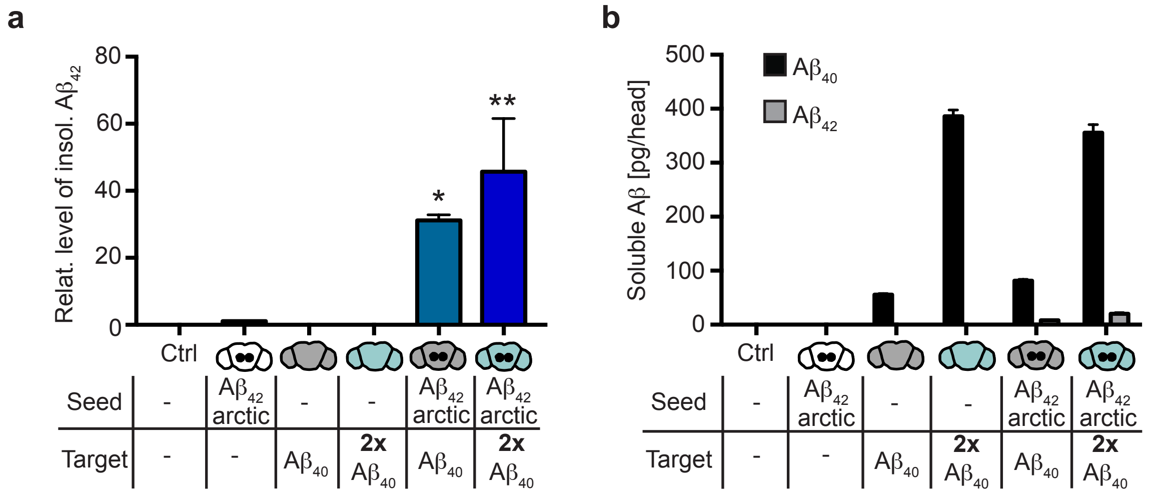

Supplementary Figure 4 | Accumulation of Aβ_{42} in *Drosophila* seeding models. (**a**) ECL measurement of insoluble Aβ_{42} in flies aged 21 days. Levels were normalized to Aβ_{42}arctic only (error bars, s.e.m., n=3 independent biological replicates, one-way ANOVA (Dunnett's multiple comparisons test), *P=0.0253, **P=0.0016). (**b**) Levels of soluble Aβ_{40} (black) and Aβ_{42} (grey) were measured using ECL assays (error bars, s.e.m., n=3 independent biological replicates).
